# Supplementary material for: Point-of-care ultrasound (POCUS): Assessing patient satisfaction and socioemotional benefits in the hospital setting
Source: PLoS One. 2024 Feb 16;19(2):e0298665. doi: 10.1371/journal.pone.0298665 (PMC10871481; doi:10.1371/journal.pone.0298665)
Supplement: S1 Table — 1Patient satisfaction survey utilized a 1–5 Likert scale, with 5 being ’very satisfied’ and 1 being ’very dissatisfied.’ Adapted from Howard et al., 2014 [3]. 2Bedside ultrasound survey utilized a 1–5 Likert scale, with 5 being ’strongly agree’ and 1 being ’strongly disagree.’ 3The responses to these survey questions were averaged to calculate the “magnitude of PPCE” variable. 4Provider competence survey utilized a 1–5 Likert scale, with 5 being the highest and 1 being the lowest. Adapted from Claret et al., 2016 [4]. 5Provider empathy survey required replacing "Dr. X" with the provider’s name and utilized a 1–7 Likert scale, with 7 being ’strongly agree’ and 1 being ’strongly disagree.’ Adapted from Hojat et al., 2011 [12]. The responses to these survey questions were averaged to calculate the “provider empathy” variable. (DOCX) [file pone.0298665.s004.docx]

|  | |
| --- | --- |
| Variables | *Median (IQR)* |
| POCUS duration (minutes) |  |
| Actual | 17.0 (9.0–25.0) |
| Patient-estimated | 10.0 (7.5–20.0) |
| Δ Time (patient) | -5.0 (-10.0– -2.0) |
| Patient satisfaction survey^1^ |  |
| “Could you rate the quality of your rapport with your ultrasound provider?” | 5.0 (5.0–5.0) |
| "How satisfied are you with the care you received at NYP?" | 5.0 (4.0–5.0) |
| "How satisfied are you with the knowledge of the providers caring for you?" | 4.0 (4.0–5.0) |
| "How would you rate the interactions you have had with your healthcare providers?" | 5.0 (4.0–5.0) |
| "How satisfied are you with the skills/abilities of the providers caring for you?" | 5.0 (4.0–5.0) |
| Bedside ultrasound survey^2^ |  |
| "The bedside ultrasound improved the interaction I had with my healthcare providers"^3^ | 5.0 (4.0–5.0) |
| "The bedside ultrasound I had was painful or uncomfortable" | 1.0 (1.0–1.0) |
| "The bedside ultrasound improved my overall satisfaction with the care I received at NYP"^3^ | 5.0 (4.0–5.0) |
| "The bedside ultrasound made my care more efficient or faster"^3^ | 5.0 (4.0–5.0) |
| "I would like a bedside ultrasound performed if I returned to the hospital in the future" | 5.0 (5.0–5.0) |
| Provider competence survey^4^ |  |
| "Please rate your confidence in the provider performing ultrasound" | 5.0 (5.0–5.0) |
| "Please rate the quality of the information delivered by your healthcare providers" | 5.0 (4.0–5.0) |
| "Please rate your overall satisfaction with hospital stay" | 5.0 (4.0–5.0) |
| "Please rate the quality of your symptom management" | 5.0 (4.0–5.0) |
| "Please rate your understanding of the information delivered by your healthcare providers" | 5.0 (4.0–5.0) |
| Provider empathy survey^5^ |  |
| "Dr. X can view things from my perspective or see things as I see them" | 7.0 (6.0–7.0) |
| "Dr. X understands my emotions, feelings and concerns" | 7.0 (5.0–7.0) |
| "Dr. X is an understanding healthcare provider" | 7.0 (6.0–7.0) |
| State-trait anxiety inventory^6^ |  |
| "I feel calm" | 4.0 (3.0–4.0) |
| "I am tense" | 1.0 (1.0–3.0) |
| "I feel upset" | 1.0 (1.0–2.0) |
| "I am relaxed" | 4.0 (2.0–4.0) |
| "I feel content" | 4.0 (2.0–4.0) |
| "I am worried" | 2.0 (1.0–3.0) |
| STAI-6 summary | 9.0 (7.0–14.0) |
|  | |
|  | |
