# Supplementary material for: Point-of-care ultrasound (POCUS): Assessing patient satisfaction and socioemotional benefits in the hospital setting
Source: PLoS One. 2024 Feb 16;19(2):e0298665. doi: 10.1371/journal.pone.0298665 (PMC10871481; doi:10.1371/journal.pone.0298665)
Supplement: S2 Table — 1Spearman rs was calculated for all variables except patient’s preferred language, for which point-biserial rpb was calculated. 2Two pairs of data points was excluded from this analysis due to missing provider ratings. 3One pair of data points was excluded from these analyses due to missing observer ratings. 4Patient’s preferred language was defined dichotomously as “English” or “Non-English” for statistical analysis. (DOCX) [file pone.0298665.s005.docx]

|  |
| --- |
|  |
|  |
|  |
|  |

|  | | |
| --- | --- | --- |
|  |  |  |
|  | *r value^1^* | *p value* |
| **Assessments of POCUS encounter** |  | |
| Δ Time (patient) | – 0.37 0.06 | |
| Patient-rated discomfort of ultrasound | – 0.35 | 0.07 |
| Provider-estimated duration of POCUS encounter | + 0.35 | 0.09 |
| Patient-rated rapport in POCUS encounter | + 0.33 | 0.09 |
| Actual duration of POCUS encounter | + 0.32 | 0.10 |
| Patient-estimated duration of POCUS encounter | + 0.23 | 0.25 |
| Δ Time (provider)^2^ | + 0.19 | 0.36 |
| Observer-rated quality of POCUS images^3^ | + 0.11 | 0.60 |
| Observer-rated environment chaos in POCUS encounter^3^ | – 0.08 | 0.69 |
| Observer-rated degree of interaction in POCUS encounter^3^ | – 0.007 | 0.97 |
| **Assessments of healthcare experience** |  |  |
| Quality of information delivered by providers | + 0.36 | 0.06 |
| Understanding of information delivered by providers | + 0.04 | 0.83 |
| **Patient characteristics** |  |  |
| CCI | – 0.15 | 0.47 |
| Patient’s preferred language^4^ | + 0.12 | 0.54 |
|  | | |
