# Supplementary material for: Point-of-care ultrasound (POCUS): Assessing patient satisfaction and socioemotional benefits in the hospital setting
Source: PLoS One. 2024 Feb 16;19(2):e0298665. doi: 10.1371/journal.pone.0298665 (PMC10871481; doi:10.1371/journal.pone.0298665)
Supplement: Supplementary file 4 [file pone.0298665.s002.docx]

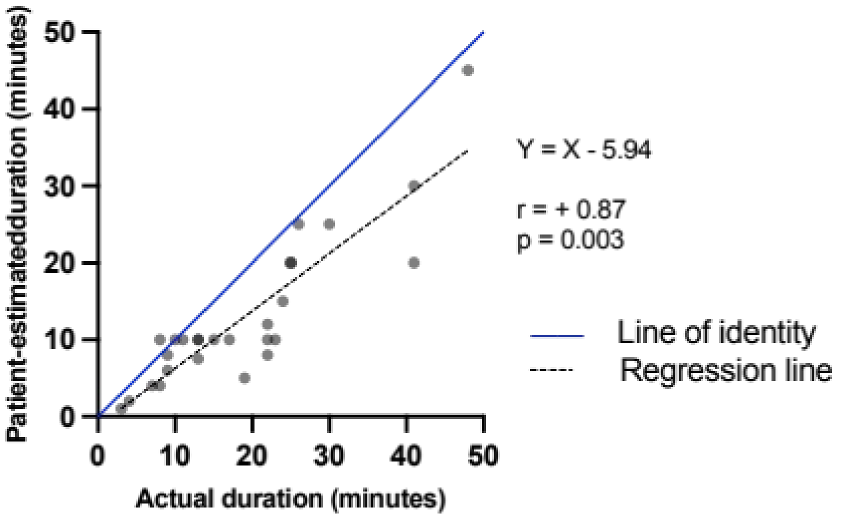

Patient-estimatedduration (minutes)
Actual duration (minutes)
Y = X - 5.94
r = + 0.87
p = 0.003
Line of identity
Regression line


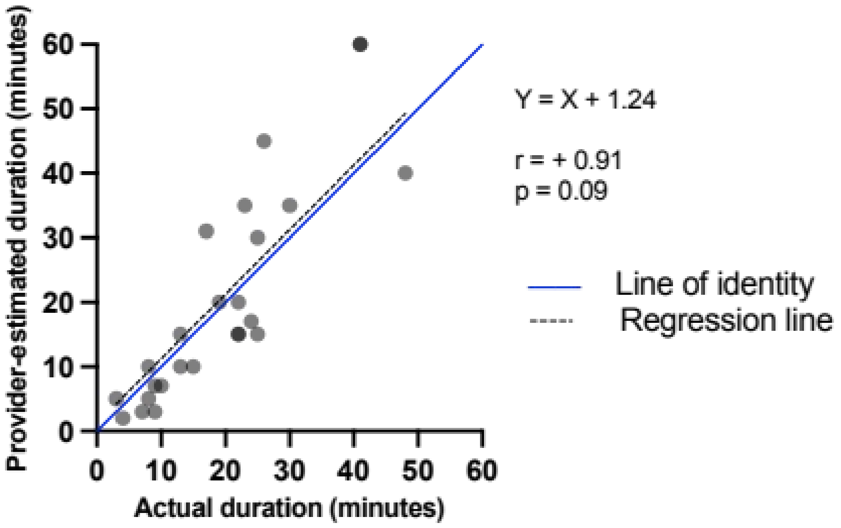

Provider-estimated duration (minutes)
Actual duration (minutes)
Y = X + 1.24
r = + 0.91
p = 0.09
Line of identity
Regression line
