# Supplementary material for: Point-of-care ultrasound (POCUS): Assessing patient satisfaction and socioemotional benefits in the hospital setting
Source: PLoS One. 2024 Feb 16;19(2):e0298665. doi: 10.1371/journal.pone.0298665 (PMC10871481; doi:10.1371/journal.pone.0298665)
Supplement: Supplementary file 5 [file pone.0298665.s003.docx]

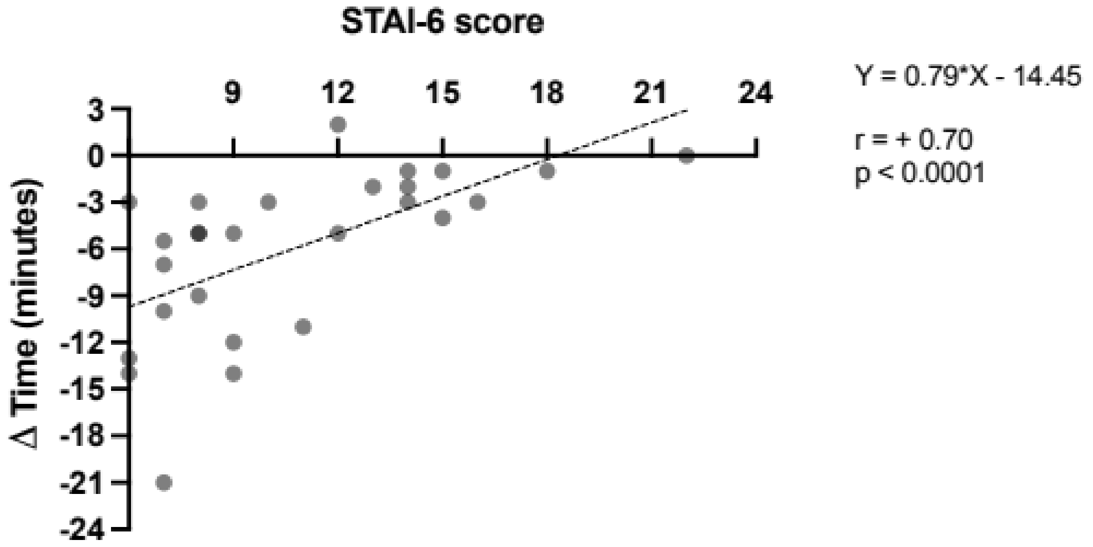

STAI-6 score
9
12
15
18
21
24
3
0
-3
-6
-9
-12
-15
-18
-21
-24
Δ Time (minutes)
Y = 0.79*X - 14.45
r = + 0.70
p < 0.0001
